# The utilization pattern of serum tumor markers in lung cancer patients: A population‐based retrospective descriptive study

**DOI:** 10.1002/jcla.23465

**Published:** 2020-07-07

**Authors:** Hongchun Wang, Jian Zhang, Xiaoli Li, Cheng Zhang, Shuli Zheng, Yihong Chi, Xia Sheng, Yi Zhang

**Affiliations:** ^1^ Department of Clinical Laboratory Qilu Hospital of Shandong University Jinan China; ^2^ Key Laboratory of Tumor Marker Translational Medicine Shandong Provincial Medicine and Health Jinan China; ^3^ IT Product and Customer Solutions Roche Diagnostics (Shanghai) Limited Shanghai China

**Keywords:** lung cancer, real‐world‐data, serum tumor marker, utilization

## Abstract

**Background:**

The trends in usage of tumor markers, including CEA, SCC, NSE, Cyfra21‐1, and ProGRP, in Chinese lung cancer patients in the real‐world setting are not fully investigated.

**Methods:**

A retrospective descriptive study was conducted using the database of Qilu Hospital of Shandong University, China between January 2013 and December 2017, involving patients primarily diagnosed with NSCLC or SCLC. Utilization trends by first discharge year, utilization rates within different durations before and after first discharge date, and combined utilization patterns of multiple tumor markers were analyzed.

**Results:**

The utilization of all these tumor markers showed increased from 2013 to 2017. CEA, Cyfra21‐1, and NSE were the most frequently detected, which increased slightly from around 50% in 2013 to around 78% in 2017 in NSCLC and from around 70% in 2013 to around 92% in 2017 in SCLC. CEA, Cyfra21‐1, and NSE were the most commonly measured within 3 months before first diagnosis with approximately 65% in NSCLC and 80% in SCLC, and ProGRP had the lowest utilization (around 30%). CEA, NSE, and Cyfra21‐1 had the highest utilization rates after first diagnosis with both around 80% in NSCLC or SCLC. Combined usage of five tumor markers was ranked the first pattern in combined utilization.

**Conclusions:**

This study suggests CEA, Cyfra21‐1, and NSE are the most frequently detected before or after first diagnosis of NSCLC or SCLC. However, SCC and ProGRP tests appeared to have relatively low usages. The utilization pattern was consistent with recommendations of guideline, but underutilization still existed.

## Graphical abstract

This retrospective descriptive study analyzed utilization of five tumor markers, CEA, Cyfra21‐1, NSE, ProGRP, and SCC in lung cancer patients.

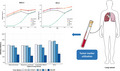

## 1 INTRODUCTION

Lung cancer is one of the most common cancers globally, accounting for an estimated 2.09 million new cases and 1.76 million deaths in 2018, ranking first among all cancer types regardless of incidence and mortality.^1^ Based on data from 339 cancer registries in China, the age‐standardized incidence rate of lung cancer was 36.71 per 100 000 and the age‐standardized mortality rate for lung cancer was 28.49 per 100 000 in China.^2^ It is estimated that lung cancer mortality in China may increase by approximately 40% between 2015 and 2030.^3^

Tumor markers are biomarkers found in blood, urine, or body tissues that can be elevated by the presence of one or more types of cancer.^4^ They contribute usefully to patient management including to guide treatment decisions, to verify the effect of treatment, to predict the chance of recovery, to predict or watch for recurrence, to diagnosis of specific tumor types, and to screen for common cancers on a population basis.^5^ Commonly used primary lung cancer markers that are currently recommended by the American Association for Clinical Biochemistry and the European Group on Tumor Markers include carcinoembryonic antigen (CEA), neuron‐specific enolase (NSE), cytokeratin fragment (Cyfra21‐1), pro‐gastrin‐releasing peptide (ProGRP), and squamous cell carcinoma antigen (SCC).^6^ The combined detection of these tumor markers can improve the sensitivity and specificity of assessments in clinical practice. They are selectively detected in auxiliary diagnosis, efficacy monitoring, and follow‐up.^7^ According to the National Academy of Clinical Biochemistry Guidelines for the use of Tumor Markers in Lung Cancer, recommended markers are Cyfra21‐1 and CEA before therapy and Cyfra21‐1 and/or CEA in post‐therapy follow‐up in patients with adenocarcinoma, squamous cell carcinoma, and large cell carcinoma. In SCLC patients, recommended markers are NSE and ProGRP before therapy and NSE and/or ProGRP in post‐therapy follow‐up.^8^

One recent study described a retrospective analysis to evaluate the frequency of serum tumor marker use in patients with advanced solid tumors.^9^ They found that a high rate of serum tumor marker testing use, and carbohydrate antigen 19‐9 (CA199) and CEA were the most commonly overused tests.^9^ Another study assessed real‐world patterns of epidermal growth factor receptor (*EGFR*) testing and associated treatment and outcomes among non‐small cell lung cancer (NSCLC) population.^10^ They found that relatively low rate of NSCLC patients received EGFR testing and significant disparities in testing were observed by different patient characters.^10^ However, relevant evidence on usage of lung cancer‐related tumor markers was lack in Chinese lung cancer patients in routine clinical practice, and whether this pattern was consistent with guideline recommendations for tumor markers in lung cancer is unknown. In the current study, we explored the trends in usage of lung cancer‐related tumor marker testing in patients with NSCLC and small cell lung cancer (SCLC) in routine clinical practice.

## 2 METHODS

### 2.1 Study design and setting

We conducted a descriptive study in Shandong province, China using clinical Laboratory Information System (LIS) database and Hospital Information System (HIS) database in Qilu Hospital of Shandong University. Qilu Hospital is a Grade A comprehensive hospital located in Shandong province and established in 1890, which was the top 20 hospitals in China. In 2018, the Qilu Hospital had approximately 3 800 000 annual outpatient and emergency visits, 210 000 annual hospitalizations, and 97 000 annual procedures.

### 2.2 Data source

Data were obtained from laboratory and hospital information systems, a real‐time system that electronically captures administrative data, clinical data, and laboratory data on patients visiting the Qilu hospital. The systems store routinely collected healthcare data from 2008 to current, which includes demographic data, hospitalization data, laboratory data, prescription data, procedure data, and imaging data. Each patient attended to the hospital was allocated a unique identifying number, which could link individual records across multiple systems.

The HIS database stores hospital discharge data, which contains some demographic characteristics of hospitalized patients, principal conditions, major medical procedures, pathology diagnosis, and hospitalization outcomes. Medical diagnostic information has been coded according to the International Classification of Disease, Tenth Revision (ICD‐10). Clinical laboratory database is held and maintained by clinical laboratory department, which contain biochemical, hematology, microbiology, virology, and serology data. Laboratory database can be record‐linked to HIS database for each patient encounter using unique patient identifier. Data were anonymized for the purposes of research that follows national healthcare big data standards, safety, and service management approach. This project was approved by Qilu hospital committee on research medical ethics.

### 2.3 Study population

All patients aged 18 years or older visited the Qilu hospital between January 1, 2013, and December 31, 2017, were eligible for inclusion. Patients may have had multiple visits to the hospital during the study period. All visits were included.

### 2.4 Study subjects

Study subjects were those with a primary diagnosis of lung cancer between January 2013 and December 2017. They were identified from HIS database with coded C34.0, C34.1, C34.2, C34.3, C34.8, C34.9 according to ICD‐10.^11^ Patients were categorized into NSCLC and SCLC patients based on pathology records. NSCLC patients were further classified into adenocarcinoma, squamous cell carcinoma, large cell carcinoma, and undifferentiated NSCLC according to pathology records.

### 2.5 Variables

Administrative data, clinical data, and laboratory data were extracted from data source. Age at diagnosis was defined as from the date of first discharge from hospital with primary diagnosis of NSCLC or SCLC to the date of birth. First discharge year was defined as the year of first discharge from hospital with primary diagnosis of NSCLC or SCLC. Length of stay (LOS) was defined as from the date of patient admission to the date of patient discharge from the hospital in each hospitalization. Observation window of tumor marker utilization was defined according to cutoffs of observation windows recommended by guideline.^7^ The utilization rate of tumor marker in one observation window was calculated as numbers of patients with one tumor marker testing divided by numbers of patients who were in follow‐up during this observation window. The frequency of combined testing of tumor markers was observed in three periods, which include the whole study period, the period of before first diagnosis of NSCLC or SCLC, and the period of after first diagnosis of NSCLC or SCLC.

### 2.6 Statistical methods

Data were summarized as mean (SD) for continuous variables and number of subjects (percentage) for categorical variables. Patient‐related demographic and clinical characteristics, utilization distribution of each tumor marker, and combined utilization distribution of multiple tumor markers were demonstrated by standard descriptive statistics. Utilization frequencies and rates within different observation windows were expressed by standard descriptive statistics. All analyses were carried out using R 3.5.1.

## 3 RESULTS

The lung cancer cohort consisted of 3443 NSCLC patients and 489 SCLC patients. Among NSCLC and SCLC patients, the majority were male, aged 45‐64 years old, with only once hospitalization, and with LOS from 8 to 14 days (Table 1).

**Table 1:** Demographic and clinical characteristics of patients diagnosed with NSCLC or SCLC

Characteristic	NSCLC		SCLC	
	number	%	number	%
No. of subjects	3443	‐	489	‐
Age at 1st discharge (continuous)^b^	62 (10)	‐	60 (10)	‐
Age at 1st discharge (y)				
18‐44	190	5.52	34	6.95
45‐64	1932	56.11	289	59.10
65‐74	1073	31.16	137	28.02
≥75	248	7.20	29	5.93
Gender				
Female	1235	35.87	141	28.83
Male	2208	64.13	348	71.17
Year of first lung cancer diagnosis				
2013	448	13.01	55	11.25
2014	675	19.60	101	20.65
2015	852	24.75	138	28.22
2016	722	20.97	103	21.06
2017	746	21.67	92	18.81
Hospitalization times (continuous)^b^	3.37 (3.8)	‐	5.12 (4.8)	‐
Hospitalization times				
1	1737	50.45	157	32.11
2	410	11.91	58	11.86
3‐5	611	17.75	90	18.40
6‐10	481	13.97	116	23.72
>10	204	5.93	68	13.91
Hospitalization days (continuous)^b^	10.8 (8.6)	‐	10.64 (7.9)	‐
Average hospitalization days				
≤7	473	13.74	45	9.20
8‐14	1488	43.22	323	66.05
15‐30	1343	39.01	113	23.11
>=31	139	4.04	8	1.64
Comorbidities				
Hypertension	434	12.61	92	18.81
Respiratory diseases^a^	441	12.81	79	16.16
Chronic ischemic heart disease	193	5.61	35	7.16
Type 2 Diabetes	168	4.88	31	6.34
Renal disease	36	1.05	4	0.82
Liver function abnormality	75	2.18	24	4.91

^a^ Respiratory diseases include acute upper respiratory tract infection, bacterial pneumonia, pneumonia, bronchitis, chronic bronchitis, emphysema, chronic obstructive pulmonary disease, bronchiectasis with infection, respiratory conditions, cryptic organizing pneumonia, other pleural conditions, acute respiratory failure, other respiratory disorders.

^b^ Data expressed as mean (SD).

**Figure 1:**
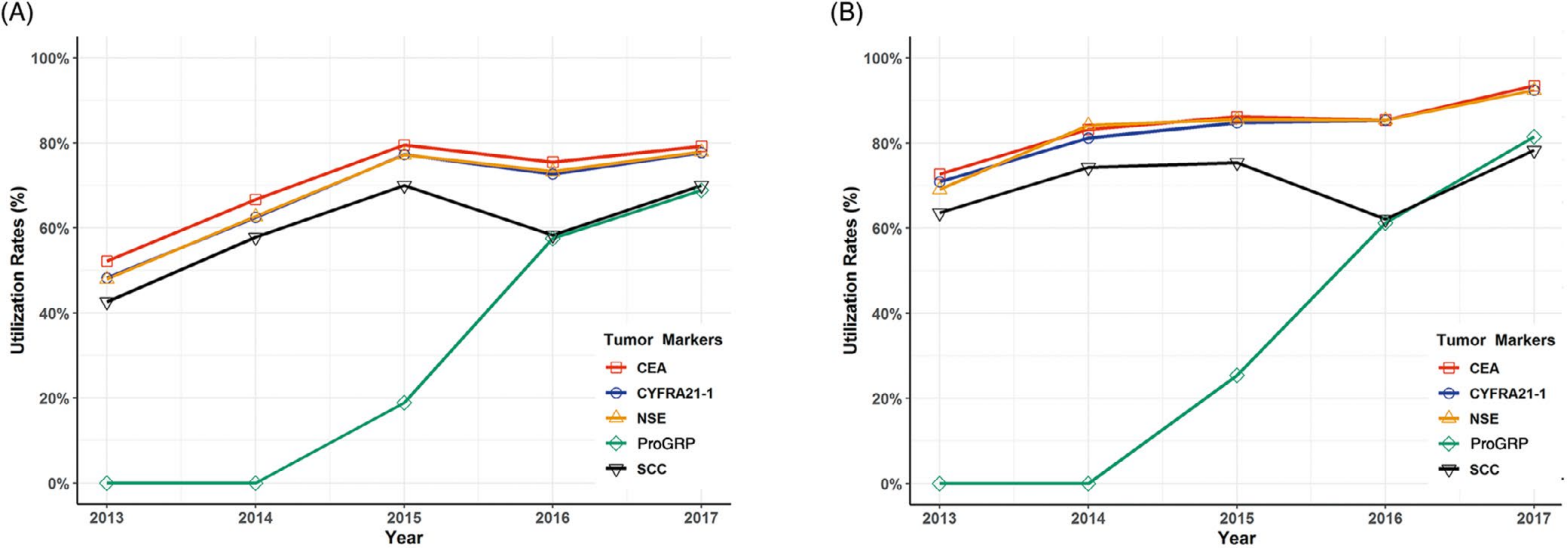
Utilization rates of tumor markers among lung cancer patients admitted to the hospital between 2013 and 2017 (A) utilization trend of tumor markers in NSCLC patients (B) utilization trend of tumor markers in SCLC patients

In NSCLC and SCLC patients, tumor marker utilization showed increased trends from 2013 to 2017 (Figure 1). CEA, Cyfra21‐1, and NSE had similar utilization rates and increased slightly from around 50% in 2013 to around 78% in 2017 in NSCLC patients (Figure 1A) and from around 70% in 2013 to around 92% in 2017 in SCLC patients (Figure 1B). Also, the utilization rate of ProGRP increased dramatically from 19% in 2015 to 69% in 2017 for NSCLC (Figure 1A) and from 25% in 2015 to 82% in 2017 for SCLC (Figure 1B). SCC utilization rates were from 43% in 2013 to 70% in 2017 in NSCLC (Figure 1A) and from 64% to 78% in SCLC (Figure 1B), but both had a slight drop in 2016.

**Figure 2:**
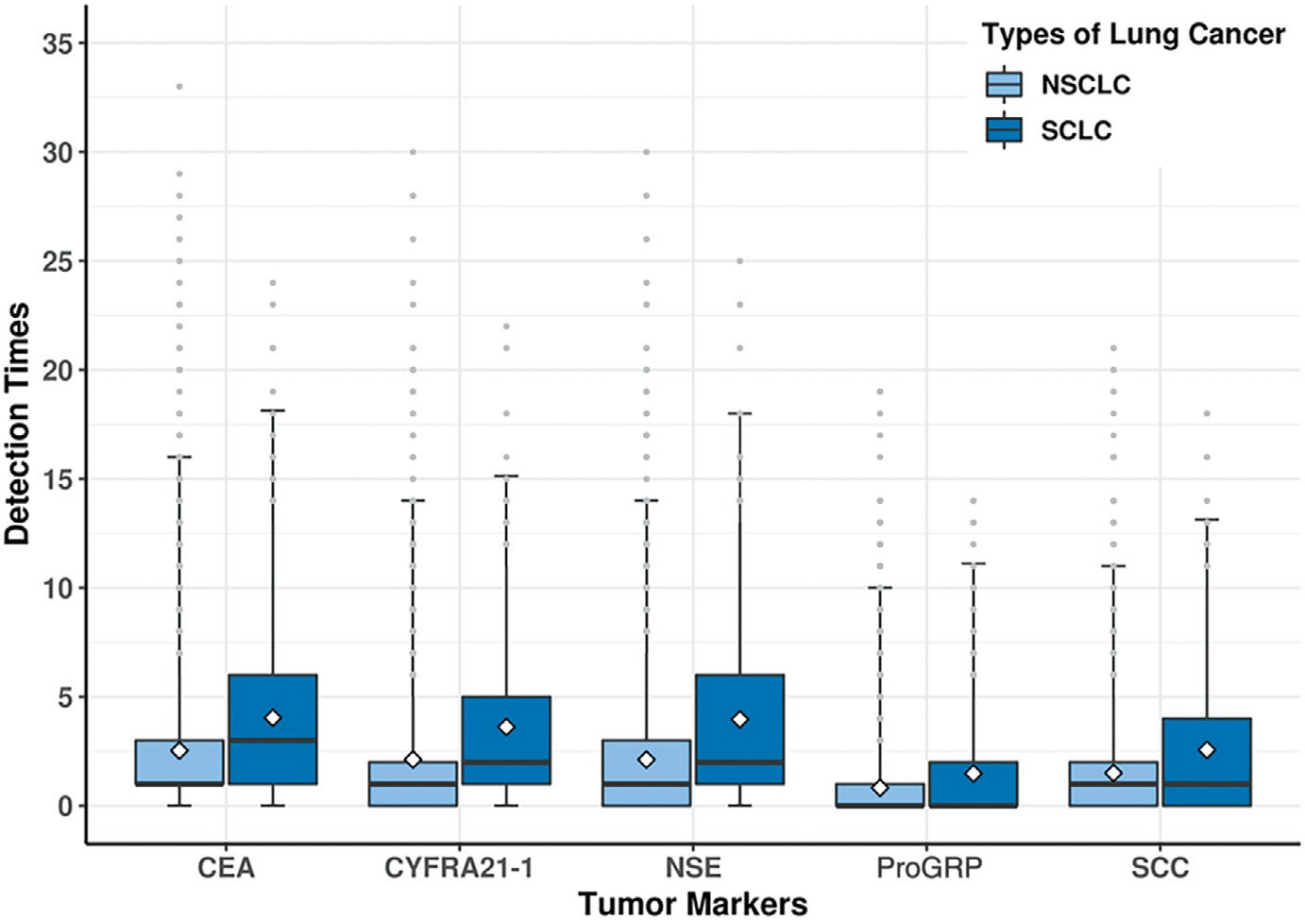
Test times for each tumor marker among patients with NSCLC or SCLC during the study period

Among patients with NSCLC and SCLC, the average test time during the study period was 2.53 and 4.04 for CEA, 2.12 and 3.61 for Cyfra21‐1, 2.12 and 3.96 for NSE, 1.50 and 2.56 for SCC, and 0.83 and 1.48 for ProGRP, respectively (Figure 2).

The utilization rate and test times of tumor markers before 3 months before first discharge of NSCLC and SCLC patients were displayed as Table 2 and 3, respectively. Generally, both the utilization rate and test times of tumor markers were increasing from 2013 to 2017, and SCLC patients had higher utilization rate and more test times than NSCLC patients. CEA, Cyfra21‐1, and NSE were obviously more utilized than ProGRP and SCC in both NSCLC and SCLC patients.

**Table 2:** Utilization rates and test times for tumor markers before 3 mo before first diagnosis of NSCLC from 2013 to 2017

Tumor markers	Index year	Utilization rate (%)	Test times (mean, min, max)
CEA	2013	3.8	3 (1,5)
CEA	2014	12.1	2.9 (1,9)
CEA	2015	11.9	2.8 (1,9)
CEA	2016	19.9	3.7 (1,18)
CEA	2017	14.2	2.7 (1,11)
CYFRA21‐1	2013	3.6	3.4 (2,5)
CYFRA21‐1	2014	7.7	2.3 (1,9)
CYFRA21‐1	2015	9.3	2.5 (1,8)
CYFRA21‐1	2016	15.9	3.1 (1,14)
CYFRA21‐1	2017	10.9	2.4 (1,10)
NSE	2013	4.0	3.5 (1,7)
NSE	2014	10.1	3.28 (1,16)
NSE	2015	11.5	3.9 (1,16)
NSE	2016	19.4	4.7 (1,28)
NSE	2017	12.9	3.4 (1,20)
ProGRP	2016	1.4	1.1 (1,2)
ProGRP	2017	4.0	2.1 (1,8)
SCC	2013	1.6	2.7 (1,5)
SCC	2014	3.9	1.4 (1,2)
SCC	2015	6.8	2.1 (1,8)
SCC	2016	7.6	1.9 (1,8)
SCC	2017	8.2	2.0 (1,8)

**Table 3:** Utilization rates and test times for tumor markers before 3 mo before first diagnosis of SCLC from 2013 to 2017

Tumor markers	Index year	Utilization rate (%)	Test Times (mean, min, max)
CEA	2013	14.5	2 (1,3)
CEA	2014	13.9	2.8 (1,4)
CEA	2015	29.0	4.4 (1,10)
CEA	2016	44.7	5.8 (1,8)
CEA	2017	51.1	5.9 (1,10)
CYFRA21‐1	2013	14.5	2 (1,3)
CYFRA21‐1	2014	8.9	2.25 (1,4)
CYFRA21‐1	2015	24.6	4.25 (1,10)
CYFRA21‐1	2016	24.3	3.2 (1,7)
CYFRA21‐1	2017	50.0	5.8 (1,10)
NSE	2013	14.5	2.25 (1,3)
NSE	2014	15.8	5.5 (2,12)
NSE	2015	32.6	6.2 (3,18)
NSE	2016	42.7	8.7 (2,15)
NSE	2017	50.0	7.8 (1,20)
ProGRP	2016	1.0	1 (1,1)
ProGRP	2017	29.3	5.4 (1,10)
SCC	2013	9.1	1.7 (1,2)
SCC	2014	5.0	1.7 (1,2)
SCC	2015	11.6	3.2 (1,4)
SCC	2016	8.7	4.5 (4,5)
SCC	2017	30.4	4.7 (1,10)

**Figure 3:**
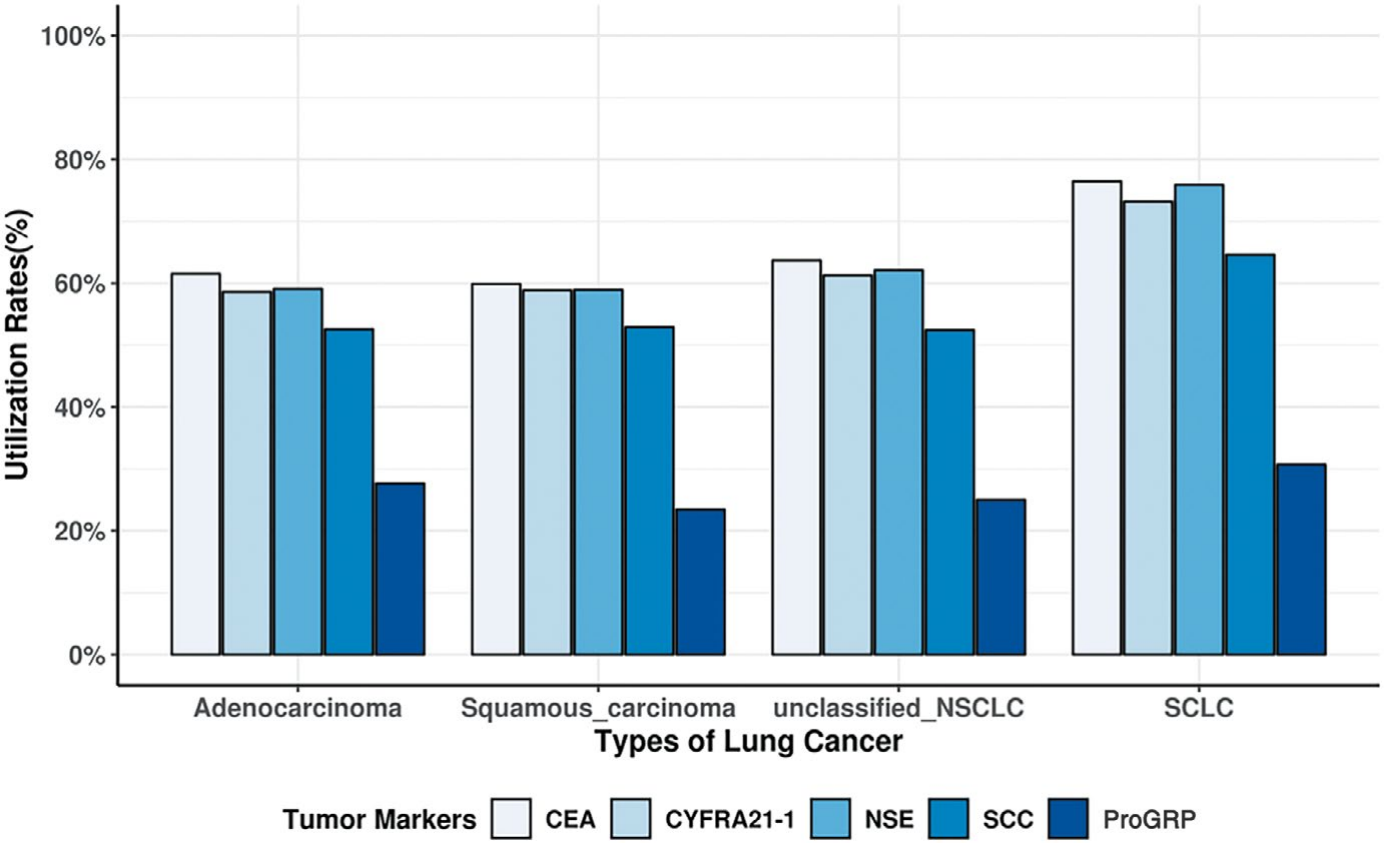
Utilization rates of each tumor marker within 3 mo before first diagnosis of NSCLC or SCLC

The usage of CEA, Cyfra21‐1, and NSE tests was similar within 3 months before first discharge with diagnosed different subtypes of NSCLC, and all utilization rates were approximately 60% (Figure 3). ProGRP test had the lowest utilization rate before first diagnosis of NSCLC. In addition, compared with NSCLC, the utilization rates within 3 months before first discharge with diagnosed SCLC were relatively high with around 75% for CEA, Cyfra21‐1, and NSE, 64.6% for SCC, and 30.7% for ProGRP (Figure 3).

**Figure 4:**
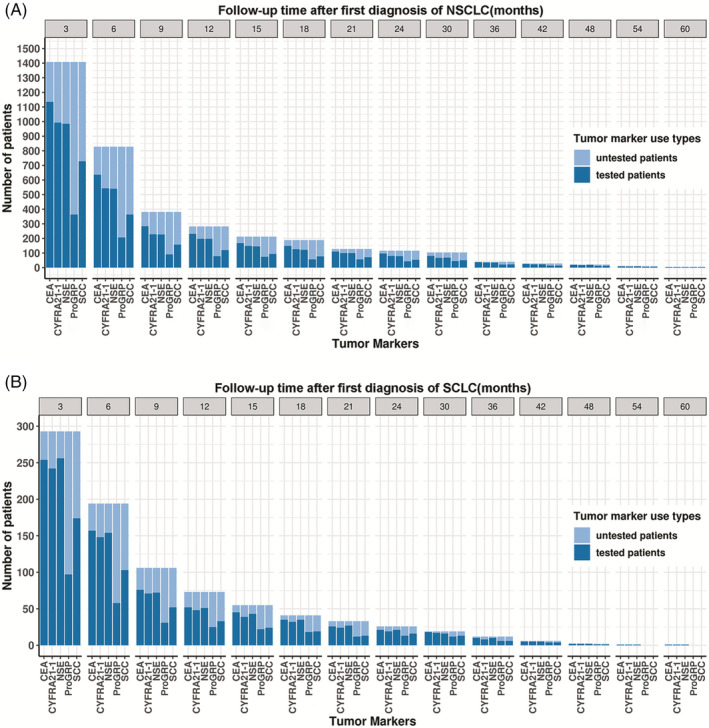
Utilization rates of tumor markers during different follow‐up durations in patients of follow‐up (A) patients with and without tumor markers testing in NSCLC and (B) patients with and without tumor markers testing in SCLC

Over 70% of NSCLC and 60% of SCLC patients had become lost to follow‐up after 3 months of first discharge from hospital with diagnosed NSCLC or SCLC (Appendix Figure S1). Among patients with follow‐up, the utilization rates of each tumor marker in different follow‐up durations were relatively stable (Figure 4). CEA, NSE, and Cyfra21‐1 were commonly detected in different follow‐up durations regardless of NSCLC (Figure 4A) or SCLC (Figure 4B).

**Figure 5:**
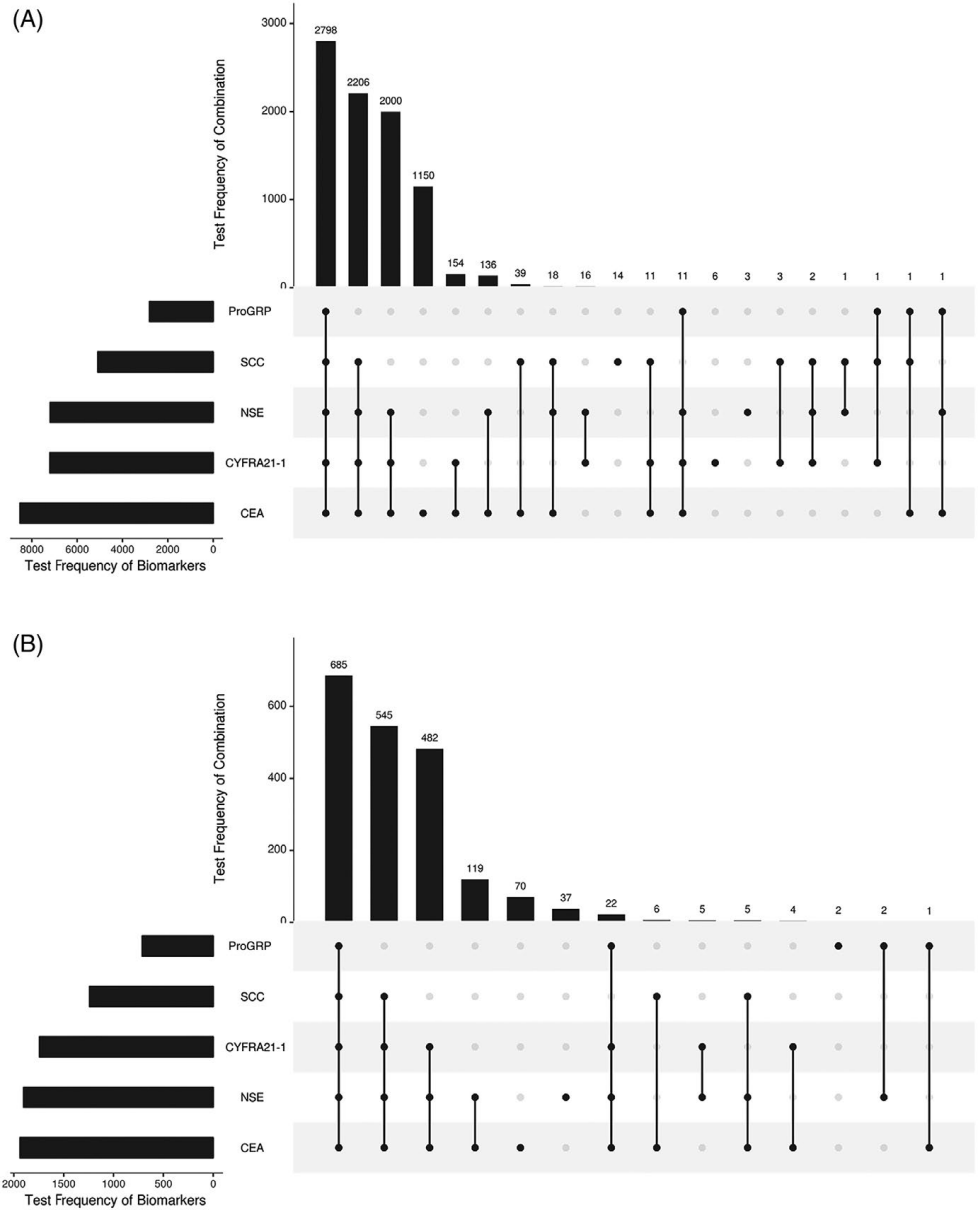
Combined utilization pattern of multiple tumor markers in patients with NSCLC or SCLC (A) combined detection frequency of tumor markers in NSCLC patients and (B) combined detection frequency of tumor markers in SCLC patients

The combined utilization pattern of five tumor markers was shown in Figure 5. The total number of hospitalizations was 11 602 in NSCLC patients and 2506 in SCLC patients. Five tumor markers were combined detection of 2798 times (24%) in NSCLC (Figure 5A) and 685 times (27%) in SCLC (Figure 5B), which was ranked the highest percentage of combined utilization pattern. The combination of CEA, Cyfra21‐1, NSE, and SCC tests was ranked the second and the combination of CEA, Cyfra21‐1, and NSE tests were ranked the third. Meanwhile, the combined utilization pattern before and after first discharge with diagnosed NSCLC or SCLC was demonstrated in Appendix Figure S2. There were 4395 hospitalizations in NSCLC and with 767 in SCLC before first discharge with diagnosed lung cancer. The most common utilization pattern of tumor markers before first discharge was combination of CEA, Cyfra21‐1, NSE, and SCC tests with the test frequency of 1174 (27%) in NSCLC (Appendix Figure S2A) and 233 (30%) for SCLC (Appendix Figure S2C). After first discharge, the total number of hospitalizations was 7207 in NSCLC and 1739 in SCLC. The combined usage of five tumor markers was the most frequently detected after first discharge with 1768 times (25%) in NSCLC (Appendix Figure S2B) and 503 (29%) in SCLC (Appendix Figure S2D).

## 4 DISCUSSION

To our knowledge, this is the first population study to demonstrate lung cancer‐related serum tumor markers utilization in Chinese lung cancer patients in routine clinical practice. The utilizations of serum tumor markers demonstrated increased trends gradually from 2013 to 2017 among patients with NSCLC or SCLC. CEA, Cyfra21‐1, and NSE were the most commonly detected before first discharge from hospital with diagnosed NSCLC or SCLC. SCC and ProGRP showed relatively low usage before first diagnosis of NSCLC or SCLC. The utilization patterns after first diagnosis of NSCLC or SCLC in different follow‐up durations were similar to those before first diagnosis. Combined usage of five tumor markers was the most common combined utilization pattern. Overall, the utilization trend of tumor markers for lung cancer was consistent with the gradual acknowledgment of their clinical importance by guideline and clinicians. However, the underutilization problem still existed in both first‐diagnosed or follow‐up patients, and this was especially true in newly included tumor marker, ProGRP.

The clinical value of tumor markers for lung cancer has greatly changed during the last decades in China. In the 2010 version of Chinese guidelines on the diagnosis and treatment of primary lung cancer, tumor markers for lung cancer were optional in qualified hospitals and only four tumor markers, CEA, Cyfra21‐1, NSE, and SCC were recommended only as references. In updated version on 2015, the importance of tumor markers in the diagnosis and therapeutic efficiency follow‐up was especially emphasized, and ProGRP was newly included by the guideline for its roles in SCLC. In the present study, we observed an increase of utilization rate of all tumor markers in lung cancer patients from Qilu Hospital of Shandong University. This trend is consistent with increasing acknowledgment for their clinical value. Furthermore, we observed a relatively lower utilization rate and also testing times of tumor markers in NSCLC patients than those in SCLC patients. We believe this difference is mostly due to the overwhelming number of NSCLC patients (n = 3443) than SCLC patients (n = 489). In clinical practices, there is a situation that a part of patients had already tested lung cancer‐related tumor marker in other hospitals and brought testing reports to doctors in paper when visiting Qilu hospital, and these results could not be included in our present study. As the incidence rate of NSCLC was greatly higher than that of SCLC, the above situation might be more common in NSCLC patients. This speculation could also be verified by the follow‐up results, in which we found that although the untested percentage of NSCLC patients was higher than that of SCLC patients in the first two discharges, the difference gradually decreased to non‐significance in the following follow‐ups.

Another significant character in our results is the low utilization of ProGRP compared with other tumor markers, and this observation could also find reasonable explanations. As mentioned above, ProGRP was only included into the recommended tumor markers in the 2015 version of guideline for the diagnosis and therapeutic efficiency testing of SCLC. With the recommendation of the guideline, the clinical laboratory of Qilu Hospital of Shandong University began to test ProGRP in 2016. This is the main explanation for the low average utilization rate of ProGRP compared with other tumor markers. However, it should be especially noticed that although the utilization rate of ProGRP had dramatically increased in 2017, its utilization rate in either first discharge or follow‐up was still lower than CEA, Cyfra21‐1, and NSE. We speculate that partly due to its relatively short history, the value of ProGRP in lung cancer had not been fully acknowledged by clinicians by the time point at which our present study ended. NSE and ProGRP are the ideal marker combination in diagnosis and therapeutic efficiency prediction of SCLC, and ProGRP showed higher specificity than NSE. According to the guideline, patients can receive further detection of tumor markers after receiving initial therapy.^7^, ^8^ Elevated level of NSE and ProGRP was observed in over 50% of patients who underwent recurrence. The recommended follow‐up frequency for tumor marker detection is every 3 months in the first 1‐3 years after treatment, every 6 months from 3 to 5 years, and every year from the fifth year. In the study, we divided follow‐up periods according to the above guideline classification. The findings still suggested that ProGRP were not well tested in SCLC patients who were in follow‐up. In recent years, ProGRP has been incorporated into the routine testing combinations for lung cancer in Qilu Hospital and with the acknowledgment of its clinical value, and the utilization rate of ProGRP would be further increased than that in 2017, which would be further testified in our future studies.

We recognize several potential limitations in our study. We could not assure whether patients with first discharge from hospital with lung cancer were diagnosed with primary lung cancer or recurrent. Previous medical information was not available for this study. Large number of patients did not attend Qilu hospital after first discharge with diagnosed lung cancer, and thus, this study could not provide meaningful results in the utilization pattern during follow‐up periods after first discharge with lung cancer. Studies in patients with good follow‐up rates are required to investigate the utilization pattern. Also, treatment information was not available for this study due to data access limitation. The lack of information has an influence on full description of the utilization pattern of these tumor markers.

In conclusion, we explored the utilization patterns of lung cancer‐related serum tumor markers in Chinese NSCLC or SCLC patients in routine clinical practice and compared the results with recommendation of Chinese guidelines on the diagnosis and treatment of primary lung cancer. Our results showed that these tumor markers showed gradually increased utilization trends over the period from 2013 to 2017. CEA, Cyfra21‐1, and NSE had similar detection rates and were the most frequently prescribed before or after first diagnosis of NSCLC or SCLC. SCC and ProGRP tests appeared to have relatively low usages in the auxiliary diagnosis or follow‐up monitoring regardless of NSCLC or SCLC. In general, the utilization pattern of tumor markers was consistent with the updates of guideline, but the utilization in either first‐diagnosed or follow‐up patients was not sufficient, especially ProGRP. Based on the above observation, we suggested the clinical importance of tumor markers in lung cancer diagnosis and treatment should be strengthened. Further studies would be conducted based on more recent data to evaluate whether the utilization of tumor markers was improved after 2017.

## Supporting information

Fig S1

Fig S2
