# Supplementary figures and images for: The utilization pattern of serum tumor markers in lung cancer patients: A population‐based retrospective descriptive study
Source: J Clin Lab Anal. 2020 Jul 7;34(11):e23465. doi: 10.1002/jcla.23465 (PMC7676212; doi:10.1002/jcla.23465)

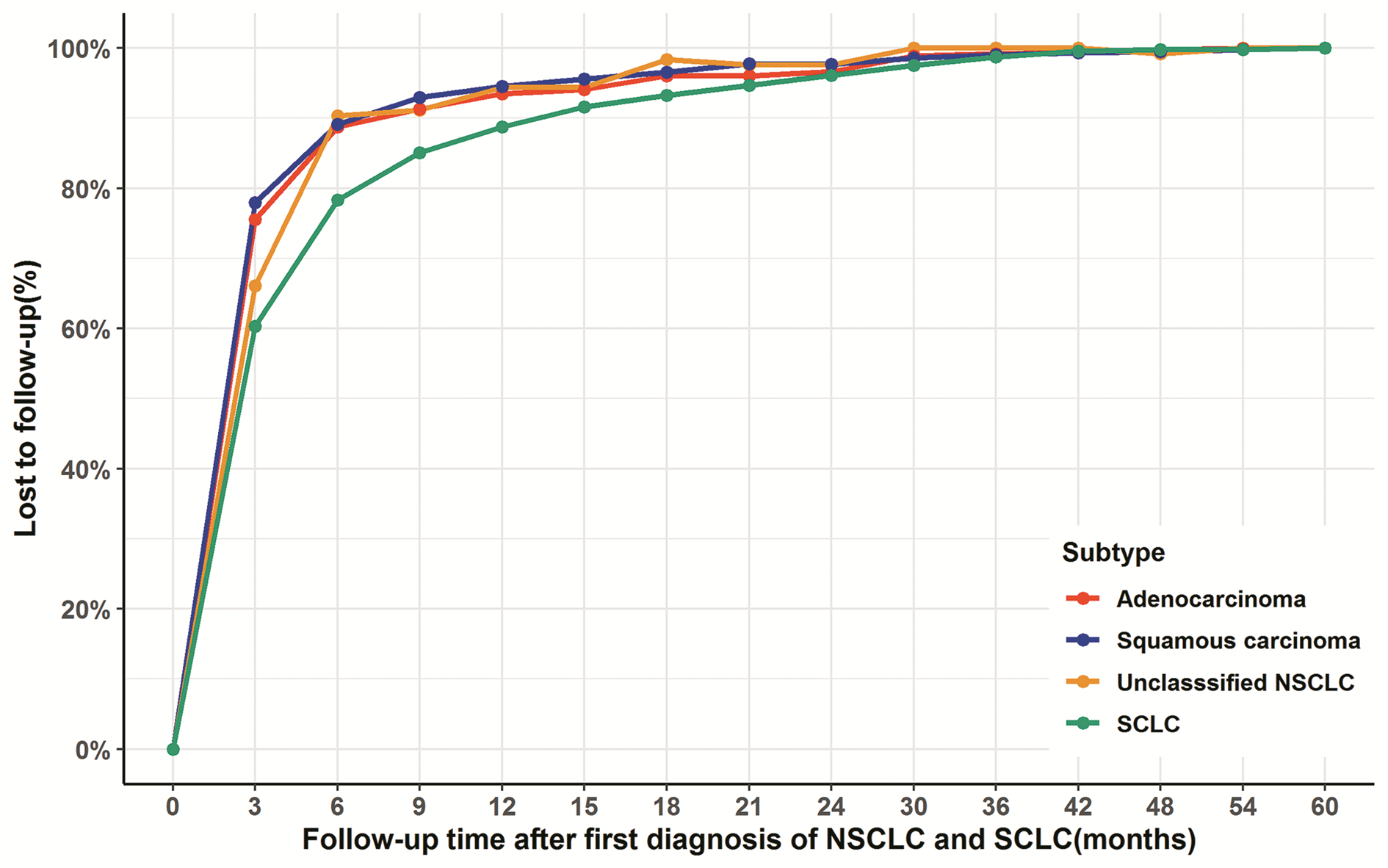

Supplement: Supplementary file 1 — Fig S1 [file JCLA-34-e23465-s001.tif]

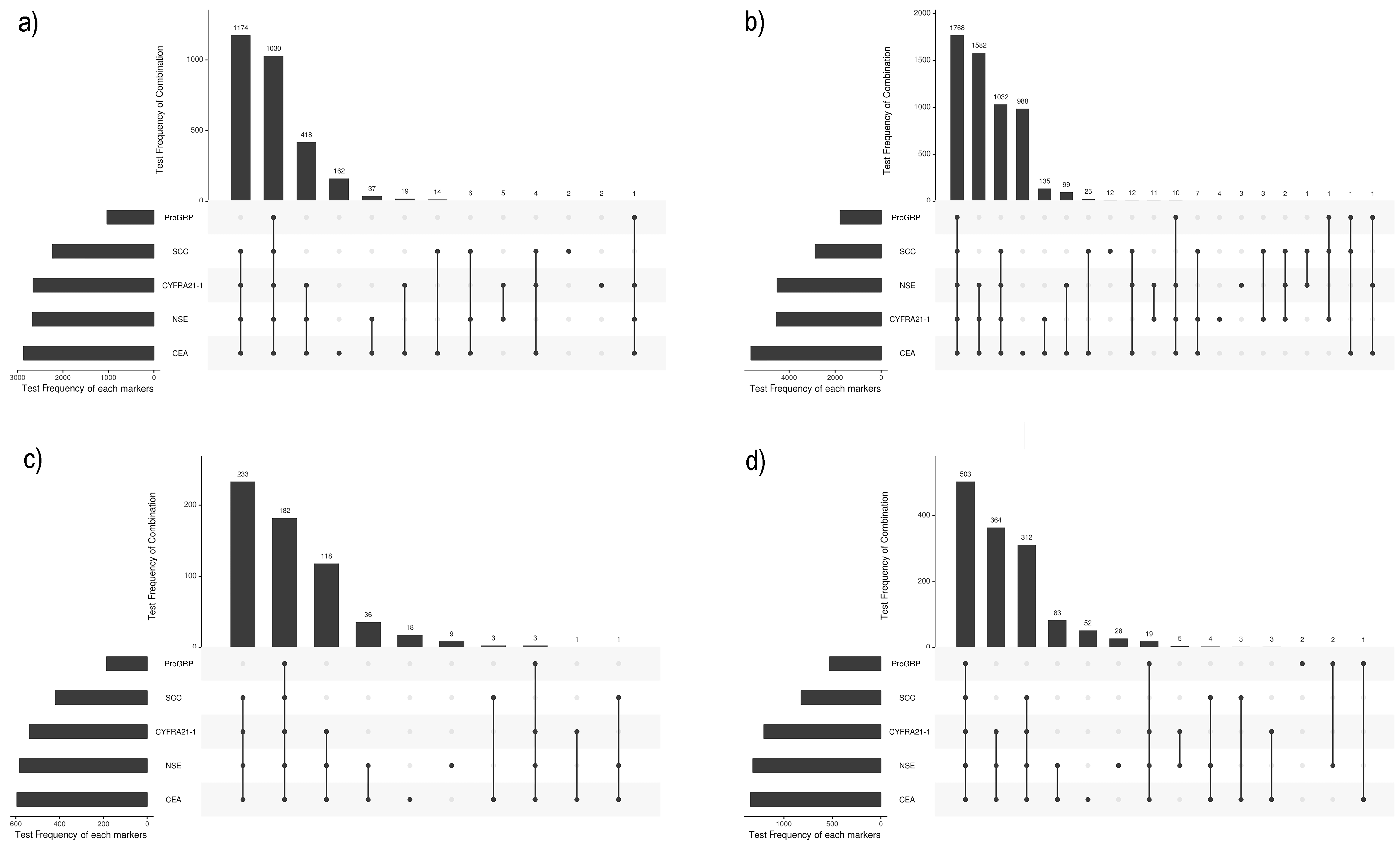

Supplement: Supplementary file 2 — Fig S2 [file JCLA-34-e23465-s002.tif]
